# The Multifaceted Role of T-Helper Responses in Host Defense against *Aspergillus fumigatus*

**DOI:** 10.3390/jof3040055

**Published:** 2017-10-04

**Authors:** Intan M. W. Dewi, Frank L. van de Veerdonk, Mark S. Gresnigt

**Affiliations:** 1 Department of Experimental Internal Medicine and Radboud Center for Infectious Diseases (RCI), Radboud University Medical Center, Geert Grooteplein Zuid 10, 6525 GA Nijmegen, The Netherlands; Intan.Dewi@radboudumc.nl (I.M.W.D.); Frank.vandeVeerdonk@radboudumc.nl (F.L.v.d.V.); 2 Faculty of Medicine Universitas Padjadjaran, Jl. Eijkman No. 38, Bandung 40161, Indonesia

**Keywords:** aspergillosis, T-helper cells, adaptive immune response, immunopathology

## Abstract

The ubiquitous opportunistic fungal pathogen *Aspergillus fumigatus* rarely causes infections in immunocompetent individuals. A healthy functional innate immune system plays a crucial role in preventing *Aspergillus*-infection. This pivotal role for the innate immune system makes it a main research focus in studying the pathogenesis of aspergillosis. Although sometimes overshadowed by the innate immune response, the adaptive immune response, and in particular T-helper responses, also represents a key player in host defense against *Aspergillus*. Virtually all T-helper subsets have been described to play a role during aspergillosis, with the Th1 response being crucial for fungal clearance. However; morbidity and mortality of aspergillosis can also be partly attributed to detrimental immune responses resulting from adaptive immune activation. Th2 responses benefit fungal persistence; and are the foundation of allergic forms of aspergillosis. The Th17 response has two sides; although crucial for granulocyte recruitment, it can be involved in detrimental immunopathology. Regulatory T-cells, the endogenous regulators of inflammatory responses, play a key role in controlling detrimental inflammatory responses during aspergillosis. The current knowledge of the adaptive immune response against *A. fumigatus* is summarized in this review. A better understanding on how T-helper responses facilitate clearance of *Aspergillus*-infection and control inflammation can be the fundamental basis for understanding the pathogenesis of aspergillosis and for the development of novel host-directed therapies.

## 1. Introduction

Infections with *Aspergillus spp*. have emerged as important opportunistic fungal pathogens in patients with a severely compromised immune system such as those receiving solid organ transplant (SOT) [1,2] and hematopoietic stem cell transplantation (HSCT) [3]. A fully functional innate immune system is required to prevent the conidia, which are inhaled on a daily basis, from causing life-threatening infections. In particular, the lack of innate effector cells, such as neutrophils, and pleiotropic impairment of immune activation by immunosuppressive therapies such as corticosteroids, are the most important factors predisposing patients to invasive aspergillosis [4,5,6,7]. Therefore, the innate immune response has been a point of major interest in the study of the pathogenesis of aspergillosis [8,9,10,11,12,13,14]. Nevertheless, recently it has become apparent that the adaptive immune response, and in particular T-helper responses also play a crucial role in pulmonary host defense [15] and the pathogenesis of aspergillosis [16]. The primary role of T-helper responses in pulmonary host defense is to augment and organize the innate immune response to deal more efficiently with invading pathogens. However, morbidity and mortality of aspergillosis can also be partly attributed to immunopathology resulting from exaggerated adaptive immune activation.

This review describes how T-helper responses are induced during host defense against *Aspergillus spp.* and in particular the most common species *A. fumigatus*. Distinct T-helper subsets and their roles in protective immunity against *Aspergillus* will be reviewed, followed by their role in allergic responses and detrimental immunopathology. Finally, it is discussed how immune-based therapies can make use of features of the adaptive immune system and/or its effector functions to improve the outcome of aspergillosis. 

## 2. Induction of T-Helper Responses by the Innate Immune System

The activation and expansion of T-helper subsets cannot be exclusively induced by the infection alone and requires innate immune mechanisms. Following inhalation, *Aspergillus* conidia encounter a wide variety of innate immune barriers in the lung.

The airway epithelial cells represent the most prominent barrier against infection; they play a major role in recruitment, activation and skewing of T-cell subsets by influencing the inflammatory cytokine environment [17,18,19,20]. Resident alveolar macrophages (AM) engulf conidia and release different cytokines and chemokines, including TNFα, MIP1-α, IL-1β, IL-1α, IL-6, G-CSF and GM-CSF [21], leading to further recruitment of innate effector cells including the dendritic cells (DCs) [9]. Monocytes also contribute significantly to the induction of T-helper responses during *Aspergillus*-infection, which is thought to be mediated through their differentiation into CD11b^+^ DCs [22]. 

**Figure 1:**
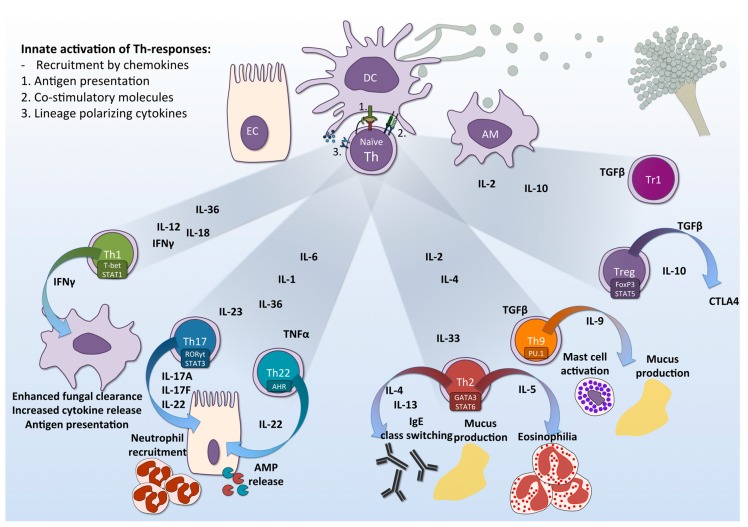
T-helper responses to *A. fumigatus.* The epithelial cell (EC), dendritic cell (DC) and alveolar macrophage (AM) constitute the innate immune response against *A. fumigatus*. Engagement of surface pattern recognition receptors (PRRs) on these cells triggers downstream signaling pathway, leading to production of distinct lineage-polarizing cytokines. This, in combination with antigen presentation by via MHC-II and binding of co-stimulatory molecules, in turn lead to activation and differentiation of naïve CD4^+^ T-helper cells to distinct effector lineages: Th1, Th17, Th22, Th2, Th9, Treg and Tr1. These effector cells differentially contribute to either protection against fungal infection, detrimental immunopathology, or in the regulation of the adaptive immune response. EC = Epithelial cell; DC = Dendritic cells; AM = Alveolar macrophages; Th = T-helper cells; IL = Interleukin; IFN = Interferon; TGF = Transforming growth factor; TNF = Tumor necrosis factor; AMP= antimicrobial peptide; CTLA-4 = cytotoxic T-lymphocyte antigen 4; STAT = Signal Transducer and Activator of Transcription, RORγt = RAR-related orphan receptor gamma t; AHR = aryl hydrocarbon receptor; t-Bet = T-box transcription factor 21; GATA3 = Transcription factor GATA-3; PU.1 = Transcription factor PU.1; FOXP3 = Forkhead box P3.

Dendritic Cells (DCs) are the key players in bridging the innate and adaptive immune response against *A. fumigatus* by specifically activating naïve CD4^+^ T-cells and triggering their differentiation into disparate lineages of effector cells [23] (Figure 1). Pulmonary DCs of mice infected with *A. fumigatus* undergo maturation upon migration, shown by increased expression of the T-cell stimulatory molecules CD80 and CD86 [24]. In addition, upon engulfment of conidia, DCs induce the migration of T-helper cells by releasing the chemokines CCL3, CCL4, CXCL10 and CCL20 [25]. Antigen presentation and T-cell activation is initiated by binding of the T-cell receptor (TCR) to major histocompatibility complex (MHC) class II, followed by interaction of co-stimulatory molecules present on the surface of T-cells and antigen presenting cells (APCs). Interaction of CD28 on T-cell surface with its ligand CD80 (B7-1) or CD86 (B7-2) on APCs and OX40 with OX40L has been shown to contribute to the immunological process of allergic forms of aspergillosis [26]. After antigen presentation and co-stimulation, autocrine production of IL-2 allows T-cells to proliferate and the cytokine milieu determines their differentiation into distinct effector cells [24,27,28,29,30] (Figure 1). The cytokine milieu is partly determined by the morphology and cell wall components of *A. fumigatus* that are recognized by the DCs [29]. Human DCs, stimulated with *A. fumigatus* conidia, trigger significant production of IL-12, which is the main cytokine inducing IFNγ-producing T-cells [27]. Different subsets of DCs exhibit distinct responses to the fungus, where monocyte-derived DC (moDC) and myeloid DC (mDC) show greatest similarities, producing pro-inflammatory cytokines IL-1β, TNFα, chemokines IL-8, CXCL1, as well as anti-inflammatory cytokines IL-4 and IL-10 specifically upon stimulation with hyphae [24,28]. TNFα release by DCs can determine whether Th17 or Th2 responses are induced leading to either neutrophil or eosinophil-mediated inflammation [30]. Infection of human DCs with *Aspergillus* hyphae but not dormant conidia results in abundant production of IL-23, subsequently inducing a Th17 response [31]. Signaling induced by cytokines binding to their complementary receptors lead to induction of lineage-specific transcription factors, that act as master regulators of distinct effector functions, helping the T-helper lineages to each exert their unique function in host defense against aspergillosis.

## 3. T-Helper Responses in Aspergillosis

Recently, all three major T-helper lineages, Th1, Th2 and Th17, have been demonstrated to play important roles during aspergillosis. *Aspergillus-*antigen specific T-cells have been found in patients as well as healthy individuals [32,33,34], and in animal models of *Aspergillus*-infection [35,36]. Inoculation with a low dose of *A. fumigatus* conidia or culture filtrate can induce resistance and protection against subsequent infection in a murine model for invasive pulmonary aspergillosis; indicating the protective role of *Aspergillus*-specific T cells in antifungal defense [36]. T-cells specific to fungal catalase and Crf1 that express CD154 and IFNγ were identified in patients recovering from invasive aspergillosis, whereas these cells were absent in patients with progressive infection [33]. These cells with a Th1 phenotype in recovering patients highlight the importance of this subset in clearing the infection [33]. 

In addition, murine models for aspergillosis have recently demonstrated that a dominant Th1 response is required for resistance to aspergillosis and induction of an efficient antifungal response [36,37,38]. Its induction relies on the Th1 inducing cytokine IL-12, which is induced in high amounts in mice that are resistant to aspergillosis [27,36,39]. IL-18, a member of the IL-1 family of cytokines is also a key player in activation of Th1 and induction of IFNγ, but only a few studies have investigated IL-18 in anti-*Aspergillus* host defense. Immunocompetent mice infected with *A. fumigatus* show elevated IL-18 in bronchoalveolar lavages and lung tissues [40], which could suggest a role for IL-18 in host defense against *Aspergillus*. However, further studies are required to elaborate the role of this cytokine in aspergillosis. The protection mediated by the Th1 subset correlates with the production of the cytokine IFNγ [36], which potentiates the fungicidal activity of innate immune cells [41,42,43,44]. Collectively, these studies emphasize the crucial role of the Th1 response in protection against *Aspergillus*, but not all T-helper responses promote clearance of the fungus from the lungs.

The Th2 response that is characterized by production of IL-4, IL-5, IL-13 and IL-10, mediates anti-inflammatory responses, allergy, and fungal persistence in the lungs [45]. The fact that a shift towards Th1 rather than Th2 is required for protection is especially highlighted in IFNγ deficient mice with an impaired protective antifungal immunity, and excessive Th2 responses [46]. A robust Th2 response is associated with a poor outcome of aspergillosis in murine models [35,46], and these responses neutralize protective Th1 responses mediated through the cytokine IL-4 [46]. In addition, IL-10 released by Th2 cells also negatively impacts protective Th1 responses in aspergillosis models, by suppressing pro-inflammatory cytokines and chemokines, inhibiting T-cell activation and IFNγ production, and promoting a Th2 response [47]. Bronchial epithelial cell stimulation with *A. fumigatus* activates protease receptor (PAR-2) and PTPN11 (SHP2), a phosphatase that inhibits IFN signaling, hence skewing the T-cell response preferably to Th2 [48]. IL-33, another IL-1 family member and a Th2-inducing cytokine, is highly expressed in mice stimulated with viable *A. fumigatus* conidia and is known to mediate immunopathology in response to chronic allergen airway exposure [49]. An elevated IL-33 level is associated with SAFS [50]. 

The mechanisms of protection against aspergillosis seemed to be very well explained by a balanced Th1 and Th2. However, with the description of the Th17 subset in 2005 [51], the understanding of how T-helper responses mediate protection against aspergillosis had to be revisited.

## 4. The Two Faces of the Th17 Response

The innate cytokines IL-1 (IL-1β and IL-1α), IL-23 and IL-6 induce Th17 differentiation by activation of the signature transcription factor of Th17 cells, retinoic acid receptor-related orphan receptor RORγT [52]. In addition, novel IL-1 cytokines IL-36α,β,γ and IL-36Ra can also regulate the induction of Th17 responses by *Aspergillus* [53]. The hallmark of Th17 cells is the production of IL-17A and IL-17F, which trigger the recruitment and activation of neutrophils to the site of infection, as well as inducing pro-inflammatory cytokines IL-6, IL-1β, G-CSF and TNFα; and chemokines CXCL8, MIP-1 and MCP1. Potentiation of neutrophils by IL-17A enhances production of ROS, proteolytic enzymes and antimicrobial peptides, altogether aiming at fungal elimination [54,55,56]. Upon activation, Th17 cells also release IL-22, which triggers epithelial cells to produce antimicrobial peptides such as β-defensin 2 and lipocalin-2 [57].

Particularly, engagement of dectin-1 on antigen-presenting cell signals through Syk/CARD9, leading to cytokine profiles that polarize naïve CD4^+^ T-cells into Th17 cells [58]. Dectin-1 mediated induction of IL-22 is crucial for releasing antimicrobial peptides that play an important role in fungal clearance [59]. Deficiency in dectin-1 is associated with a defective Th17 response, and therefore impaired neutrophil recruitment and excessive fungal growth [56,58]. On the one hand, the Th17 response is activated by stimulation of dectin-1 with β-glucans, whereas on the other hand, galactosaminogalactan (GAG) can inhibit Th17 responses in vitro and in vivo, via induction of IL-1Ra [60]. By diminishing the Th17 response, GAG was able to decrease neutrophil recruitment, thereby increasing susceptibility of WT mice to invasive aspergillosis [60,61]. Additional evidence for the importance of the Th17 response in host defense against *Aspergillus*-infection is provided by the observation that patients with *Aspergillus* skull base osteomyelitis have defects in Th17 responses [62]. PBMC from patients with chronic granulomatous disease exhibited a lower IL-17A production upon stimulation with *Aspergillus* and *Candida* compared to healthy donors, and this might contribute to ineffective fungal clearance in these patients [63]. 

Despite the important role of Th17 for fungal clearance, uncontrolled or prolonged Th17 activation is detrimental to the host, by causing pulmonary damage and persistent inflammation [64,65]. Increased Th17 responses are attributed to severe immunopathology characterized by massive neutrophil infiltrates in the lung parenchyma and impairment of fungal clearance [66]. The importance of IL-23 in maintaining the Th17 response is highlighted by the lack of IL-17 producing cells in IL-23p19 ^−/−^ mice; whereas in mice with pulmonary aspergillosis, the absence of IL-12 leads to enhanced IL-23 production and increased susceptibility to *A. fumigatus*. Neutralization of IL-23 and IL-17 can enhance antifungal resistance and decrease fungal burden in models of aspergillosis with detrimental immunopathology [66]. Furthermore, impairment of the IFNγ response is associated with increased interleukin-17a expression and attributes to mortality in mice with invasive aspergillosis [67]. The Th17 response is also partially dependent on Toll IL-R8 (TIR8)/single Ig IL-1-related receptor, a member of the IL-1R family which negatively regulates IL-1R signaling. Tir8^−/−^ mice were more susceptible to infection and exhibited a higher inflammatory pathology; this finding highlighted the possible role of TIR8 in orchestrating protective response or immunopathology against fungal infection [68]. In vitro stimulation of circulating T-cells demonstrates a preference towards an induction of a Th1 profile [43,69], however, lung derived T-cells isolated from COPD patients were found to have a preference to a Th17 phenotype [69]. Interestingly, the capacity to induce pulmonary Th17 responses to *Aspergillus* has also been associated with bacteria colonizing the gut microbiota. The presence of segmented filamentous bacteria can influence the pulmonary adaptive immune response partly by increasing Th17 cells population in the lungs [70]. In contrast to the Th17 response that has beneficial effects, the Th2 response not only suppresses protective immunity but also is the key player in allergic disease associated with *Aspergillus*. 

## 5. Th2 Response Drives Allergic Hypersensitivity Reactions to *Aspergillus*

The Th2 response is not beneficial for fungal clearance in invasive disease, moreover, a Th2 response mounted towards *Aspergillus* can lead to allergic disease in relatively healthy individuals and in individuals with underlying pulmonary disease such as asthma and cystic fibrosis. It is increasingly recognized that in a significant group of patients, their severe asthma is associated with fungal exposure [71]. There are estimates that fungal molecules represent approximately 16% of the known allergens [72,73]. To date, over 150 fungal allergens have been identified [74], with the most numerous found in *A. fumigatus* [73,75]. The response to these allergens includes a strong Th2 response that leads to the development of *Aspergillus*-specific IgE, which remains to be a diagnostic marker for severe asthma with fungal sensitization (SAFS) [76,77]. A more severe form of allergic response to *Aspergillus* is allergic bronchopulmonary aspergillosis (ABPA), which is associated with prolonged fungal exposure and typically occurs in individuals with a hypersensitive immune response, where airway inflammation, eosinophilia, and abundant production of *Aspergillus*-specific IgE are usually present [78]. This allergic form of aspergillosis occurs in 1–2% of asthmatic patients and up to 10% of patients with cystic fibrosis (CF), in the latter it is a major cause of deteriorating lung function and mortality. Bronchoalveolar lavage (BAL) of ABPA patients showed the presence of infiltrates rich in eosinophils, neutrophils, lymphocytes and often fungal hyphae [78,79].

**Figure 2:**
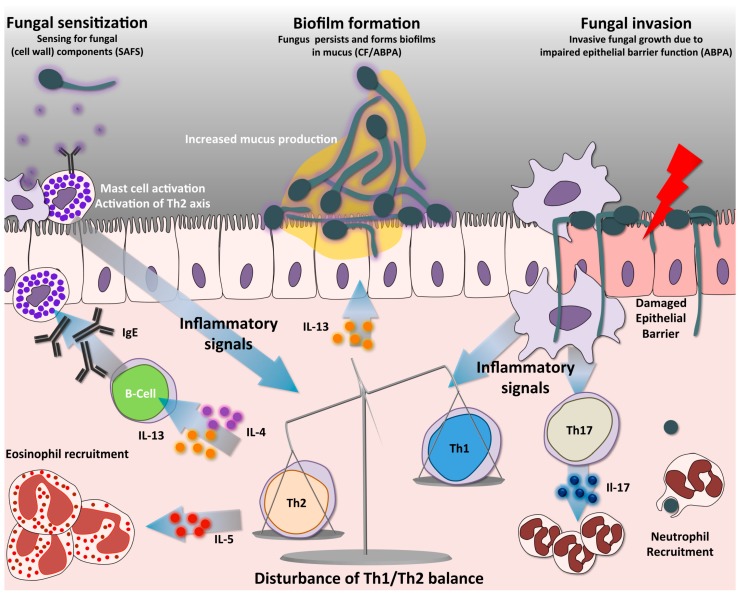
Immune pathways in SAFS and ABPA. Antigen presentation by DCs activates naïve CD4+ T-cells into distinct Th-cell lineages. A predominant non-protective Th2 response is a hallmark in allergic forms of aspergillosis, such as SAFS and ABPA. A distinct characteristic is that the high Th2 response creates an imbalance resulting in low protective Th1 responses. Th2 cells release different cytokines, among them IL-4 and IL-13, which trigger antibody class switching to IgE, a distinct hallmark of SAFS. In addition, these cytokines mediate increased mucus production by respiratory goblet cells, and IL-5, which triggers the recruitment of eosinophils. Abundant mucus production in the airway allows biofilm formation, thus facilitating fungal growth. Furthermore, the absence of fungal clearance leads to continuous airway sensitization with fungal components, activating mast cells and the Th2 axis. Mast cell degranulation releases abundant inflammatory mediators such as histamine and leukotriene, which also contribute to the inflammatory phenotypes of patients. Activation of Th17 cells facilitates recruitment of neutrophils, partly contributing to the persistent immunopathology of these diseases. During ABPA the pulmonary epithelial barrier can become compromised, allowing *A. fumigatus* to germinate and invade the tissues. SAFS = Severe asthma with fungal sensitization; ABPA = Allergic bronchopulmonary aspergillosis; CF = Cystic fibrosis.

The pathogenesis of these allergic forms of aspergillosis is thought to be mediated predominantly by a hyperactive Th2 response (Figure 2), leading to airway hypersensitivity, IgE production and persistent airway inflammation [45]. The hyper-reactive Th2 response can be observed in the response of Peripheral Blood Mononuclear Cells (PBMCs) of ABPA patients, who demonstrate a disturbed Th2/Th1 ratio upon stimulation with *A. fumigatus* conidia [80]. This disturbed ration is characterized by elevated IL-5 and IL-13, and a low IFNγ response in comparison to non-allergic controls [80]. Similarly, in mouse models, repeated exposure to low doses of conidia primes the development of strong Th2 and Th17 responses, resulting in chronic inflammation resembling ABPA [81]. The repetitive exposure to *A. fumigatus* increases eosinophil accumulation in the lungs and peripheral blood, airway remodeling, and elevated IgE in different murine ABPA models, which can be attributed to increased IL-4 and IL-5 and diminished IL-10 production [82,83,84]. In addition to the high Th2 response, Th17 responses also significantly contribute to the detrimental inflammatory response observed in *Aspergillus* related allergy, by stimulating excessive neutrophil recruitment [81,85,86]. Especially under pre-existing conditions where the immune response is skewed to a Th2 response such as in allergy, infection with *A. fumigatus* results in increased Th17 response, which could lead to persistent inflammation and defective fungal clearance [87]. It was recently discovered that the Th17 cytokine IL-17F is also involved in allergic airway inflammation, and this effect is mainly dependent on signaling through IL-17RC [86]. In mouse models with Th2/Th17 eosinophilic and neutrophilic allergic airway inflammation induced by *A. fumigatus* hyphal extract, administration of mesenchymal stromal cells (MSC) was shown to significantly decrease airway hyper-responsiveness, through reduction of Th17-mediated inflammation [88]. 

The Th9 subset has only recently been described [89], and is closely associated to the Th2 response. Th9 cells are known to mediate inflammation, infection and allergy, and this lineage is differentiated in the presence of Th2-polarizing cytokine IL-4, in combination with IL-2 and TGFβ [90]. Fully differentiated Th2 cells cultured with TGFβ results in increased production of IL-9, which is a signature cytokine of Th9 [90]. In healthy volunteers, the fungal pathogen *C. albicans* induced IL-9 production, mostly by IL-9^+^IL-17^+^-co-expressing CD4^+^ T cells than IL-9 single positive cells. However in patients with hyper IgE syndrome (HIES) caused by STAT3 mutation, this IL-9 response was significantly lower in the presence of IL-4, which could be due to the deficiency of Th17 cell subset in these patients [91]. The Th9 subset plays a role in the allergic response to *A. fumigatus* in cystic fibrosis, and blockade of the Th9 response in this setting could represent a novel therapeutic strategy to reduce infection associated inflammation [92].

## 6. Regulatory T-Cells

**Figure 3:**
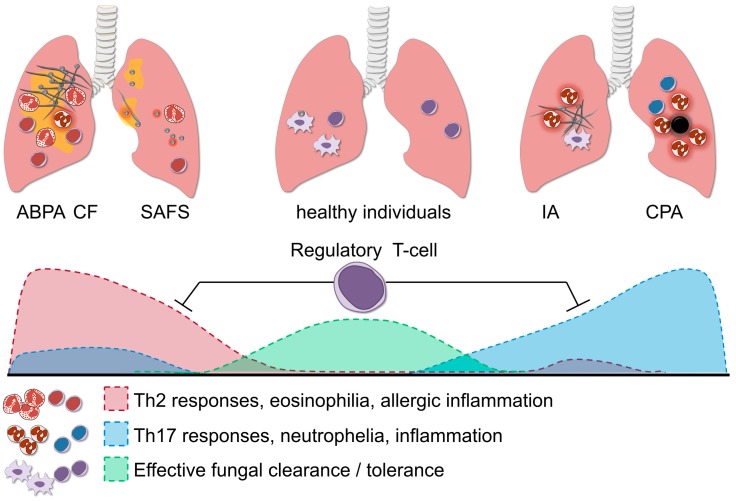
The role of regulatory T-cells in aspergillosis. Regulatory T-cells (Treg) orchestrate the balance of the Th1, Th2 and Th17 response; and this fine-tuning is necessary for effective fungal clearance in the host. In healthy individuals, the protective Th1 response effectively aids the clearance of *A. fumigatus*, whereas potential damaging excessive Th17 activation is dampened by Treg. A predominant Th2 response in allergic forms of aspergillosis such as SAFS and ABPA, leads to persistent inflammation and fungal colonization. In immunocompromised individuals, failure of innate immune cells to eliminate fungi may cause invasive aspergillosis (IA). When the Th17 response is too potently induced this might lead to excessive neutrophil influx and collateral damage. Fine-tuning of the strong pro-inflammatory responses during aspergillosis is facilitated by Treg, which in turn suppress the Th17/Th2 activity and prevent damage to the host.

Regulation of a balanced T-helper response is crucial for resolution of the inflammatory response when infection has been resolved, but also for prevention of detrimental immunopathology during infection or hypersensitivity (Figure 3). Regulatory T-cells are endogenous regulators of inflammatory response, they control inflammation by a variety of mechanisms, for example by contact dependent inhibition of other leukocytes via the surface receptor cytotoxic T-lymphocyte antigen 4 (CTLA-4) or through release of anti-inflammatory cytokines IL-10 and TGF-β [93]. Two types of regulatory T-cells (Treg) are known to play a role in the antifungal response against *A. fumigatus*. Natural Treg (nTreg) originate in the thymus and are present in the periphery, providing tolerance in early infection and limiting neutrophil activity, and induced Treg (iTreg) that are primed from naïve CD4^+^ Th cells, and these cells limit inflammation in later stages of infection, preventing fungal allergy by producing IL-10 and TGFβ [94]. Repeated exposure to aerosolized antigen in mice induces CD4^+^ T-cells expressing both surface and soluble TGFβ, and adoptive transfer of these cells to naïve recipient mice abrogates the allergic phenotype [95].

Similar to the pro-inflammatory T-cell lineages, the Treg [32,34] and Type (1) regulatory T-cells (Tr1) [34,94] cells found during aspergillosis in both humans as well as murine models show *Aspergillus-*specificity. Type (1) regulatory T-cells (Tr1) are distinct from Treg in their mode of induction, cytokine production and phenotype. *Aspergillus*-specific Tr1 are present in peripheral blood of human and mice. Vaccination with Crf1/p41, a cell wall component of the fungus, generated IL-10-producing Tr1 cells and suppressed antigen-specific T-cell proliferation [34]. Whereas in individuals with allergy to *A. fumigatus*, despite the presence of Tr1 cells, specific Th2 cells were expanded, indicating the capacity of the fungus to modulate distinct regulatory responses to prevent allergic reactions [32]. 

In individuals colonized with *A. fumigatus* that induce a strong Treg response, a high concentration of vitamin D3 in the serum was observed, whereas patients with ABPA that exhibited a predominant Th2 response had a lower vitamin D3 serum concentration [96]. In line with this, vitamin D3 supplementation was found to decrease *A. fumigatus-*specific Th2 responses in cystic fibrosis patients [97].

Regulation of Treg function *versus* immunopathology during aspergillosis was found to be closely associated with tryptophan metabolism. The enzyme Indoleamine 2, 3-dioxygenase (IDO) catalyzes the breakdown of tryptophan to kynurenine and is known to activate Treg responses, whereas the presence of IL-23 and Th17 negatively regulates it [66]. Imbalance of the Th17/Treg axis in certain conditions contributes to the pathogenesis of aspergillosis [98]. In mice with chronic granulomatous disease, lack of reactive oxygen species (ROS) disrupted the metabolism of tryptophan along the kynurenine pathway, leading to excessive production of IL-17, defective Treg function, and hyperinflammation. Supplementation with natural kynurenines were able to reverse this hyperinflammatory response [99]. Early in aspergillosis, CD4^+^CD25^+^ Treg recruitment is able to control inflammation by neutrophil suppression, mediated by the actions of IL-10 and CTLA-4 on IDO. Furthermore, IFNγ levels in this early phase of infection partially condition the subsequent adaptive immune response by inducing IDO-dependent tolerogenic DCs, which subsequently activate tolerogenic Treg that produce IL-10 and TGFβ, inhibit Th2 cells, and prevent fungal allergy [94]. IDO activation could also be activated in a TLR3/TRIF dependent manner in epithelial cells, resulting in protection against *Aspergillus*. Mice defective in this pathway exhibited a stronger Th17 response manifested by infiltration of neutrophils in the lungs, decreased Th1/Treg response, and higher fungal burden and immunopathology [20].

## 7. Immunotherapy: Optimizing the Potential of T-Helper Responses

Despite the development of strict antifungal treatment regimens, the mortality and morbidity due to aspergillosis remained relatively unchanged within the past few years [7,100]. Antifungal resistance and a persistent dysregulated immune response in patients with aspergillosis limits the efficacy of antifungal medication [101]. The immunological state of the host remains an important determinant in the outcome of infection, with the absence of efficient innate immune responses predisposing for invasive infection and the induction of hyper-inflammatory responses contributing to immunopathology. Therefore, restoration of dysregulated immune responses, by using immune-based therapies, is believed to be a promising strategy to improve the clinical outcome of fungal infections [102]. Numerous immunomodulatory therapies in pre-clinical experimental phase are aimed at distinct processes of the host-pathogen interactions [103,104,105,106,107,108]. It should be noted that most immunomodulatory strategies are aimed at boosting the adaptive immune response or at making use of the protective mechanisms provided by the adaptive immune response. 

As previously discussed, a robust Th1 response is crucial for efficient clearance of *A. fumigatus* [36,40,43]. To simulate a robust Th1 response, patients can be administered IFNγ, the signature Th1 cytokine. Administration of IFNγ was shown to have the potential to augment the host response to control infection against *A. fumigatus* in immunocompromised individuals and CGD patients, improving outcome and reducing mortality [106,107,109]. Adjunctive therapy of GM-CSF and IFNγ alongside antifungal treatment was also able to improve disease outcome in both neutropenic and non-neutropenic patients [105,110,111]. 

In contrast to strategies aiming at augmenting the host response, cytokine-based immunotherapies can also be employed to prevent detrimental immunopathology. The Th2/Th1 imbalance observed in ABPA patients could be restored in vitro using IFNγ [81], suggesting that IFNγ therapy could be a promising strategy to skew the immune responses in ABPA and fungal allergy. Interference of the IL-1 pathway has been an interesting subject in recent years. Inflammasome-dependent IL-1β production is the driving force for Th17 induction, and in certain condition such as cystic fibrosis and CGD this Th17 response can lead to exaggerated tissue damage in the host. Administration of recombinant IL-1 receptor antagonist (IL-1Ra) decreases immunopathology and contribute to a better outcome in Th17-mediated inflammation as a result of *Aspergillus*-infection in a murine cystic fibrosis model [112] and in a murine CGD model [113]. In corticosteroid immunosuppressed mice, detrimental damage leading to pulmonary tissue hypoxia could be reduced by IL-1Ra [114]. 

In immunocompromised individuals, such as those undergoing transplantation, cellular immunity is abrogated. Innate immune cell counts subsequently recover, however, lymphocyte numbers remain low for several months, which renders these patients susceptible to infections [115]. Adoptive transfer of in vitro-generated CD4^+^ T-cells in patients receiving allogeneic HSCT has been shown to improve clearance of *Aspergillus* infection and confer protection against invasive aspergillosis [104,116]. Clinical-scale generation of anti-*Aspergillus* T-cells can be achieved by stimulating T-cells with a single antigenic epitope, however, whole fungal extract generates T-cells with more efficient anti-fungal properties [116,117]. In addition to expanding normal T-cells, T-cells can also be engineered to have an improved antifungal capacity. Genetic modification of T-cells to express a chimeric antigen receptor (CAR) leads to a redirected signaling after recognition of antigens. By generating a T-cell that expresses dectin-1 that activates CD28 and CD3ζ (referred as D-CAR), a T-cell can directly recognize the fungus and induce its anti-fungal effect [118]. Adaptation of other types of receptors specific for *A. fumigatus* and further validation both in vitro and in vivo is necessary to improve the optimization and application of this promising treatment.

Although there are a growing number of experimental strategies to augment the antifungal host response by targeting T-helper responses, it should be considered that treating disease in immunocompromised patients is more challenging than experimental models, since these individuals have more complex clinical settings. Therefore, the added value of various immunotherapeutic approaches needs to be evaluated on a patient-to-patient basis. The host defense in different patients can be altered in different ways, and different underlying diseases can potentially complicate treatment. Therefore, immunotherapy requires a tailored and personalized approach, taking into account the complexity of different cases and underlying diseases. Well-targeted clinical trials with the right patients and controls aiming at applying these therapies in clinical settings are required to realize immunotherapy for *Aspergillus*-related diseases in the near future.

## 8. Future Perspectives

Most of the knowledge on T-helper responses in aspergillosis is derived from murine models. Although experimental infection models provide invaluable information on pathogenesis of aspergillosis and the role of T-helper responses, such models cannot always be inferred to humans due to differences in human and murine physiology and host defense. In addition, murine models may not always be representative for patients with aspergillosis, who present with a complex clinical background, can have various underlying diseases and host defense can be altered in multiple and interacting ways. We therefore believe that an important future direction of research should include the evaluation of T-helper responses to *Aspergillus* in humans in general and preferably in patients with aspergillosis specifically. In this way, findings from murine models can be validated and translated to patient settings, and a detailed overview of the human T-helper responses against *A. fumigatus* can be elaborated. 

Although T-helper responses are usually described as specific cell subsets, recent studies have demonstrated that in fact there is overlap between the different T-cell subsets [113,114,115,116,117,118,119,120,121,122]. Th17 cells co-expressing IFNγ or IL-10 have been found in response to the fungal pathogen *Candida albicans* [121]. However limited numbers of IL-17/IFNγ co-expressing cells were observed to be induced by *Aspergillus* in human PBMCs [122]. IL-22, however, was found to be produced not only by Th17 cells, but also in a T-cell subset co-expressing IFNγ and in T-cells exclusively expressing IL-22 [122]. Another discovery that complicates our understanding of the T-helper responses is that regulatory T-cells can co-express IL-17 and potentially contribute to detrimental immunopathology [119]. Recent studies have demonstrated that *A. fumigatus* also induces regulatory T-cells with a Th17-like phenotype [120], however, it remains to be determined to what extent these cells contribute to the detrimental immunopathology seen during aspergillosis. The observation that the expression of lineage-defining cytokines showed a great plasticity among *Aspergillus*-activated T-cells complicates our understanding of the mechanisms going on in vivo during infection. Increased knowledge and understanding of this plasticity of the T-cell response might in the future lead to strategies that are aimed at utilizing this plasticity to shape T-cell responses in a way for optimal fungal clearance and limited immunopathology. The administration of recombinant cytokines and adoptive transfer of pathogen-specific T cells are several examples of potential successful approaches that have been developed in the past few years. Fundamental studies regarding the therapeutic potential of T-cell plasticity in aspergillosis and clinical trials aiming at applying these therapies in clinical settings are required to realize immunotherapy for *Aspergillus*-related diseases in the near future.
